# Study on a Strong Polymer Gel by the Addition of Micron Graphite Oxide Powder and Its Plugging of Fracture

**DOI:** 10.3390/gels10050304

**Published:** 2024-04-30

**Authors:** Bin Shi, Guangming Zhang, Lei Zhang, Chengjun Wang, Zhonghui Li, Fangping Chen

**Affiliations:** 1 Hubei Key Laboratory of Drilling and Production Engineering for Oil and Gas, School of Petroleum Engineering, Yangtze University, Wuhan 430100, China; stonersb2@163.com (B.S.); lizhonghui2024@163.com (Z.L.); 2 Research Institute of Shaanxi Yanchang Petroleum (Group) Co., Ltd., Xi’an 710075, China; geda2024@163.com; 3 Hubei Provincial Key Laboratory of Oil and Gas Exploration and Development Theory and Technology, Department of Petroleum Engineering, China University of Geosciences (Wuhan), Wuhan 430074, China; zhangshishishi.188@163.com; 4 College of Chemistry and Chemical Engineering, Xi’an University of Petroleum, Xi’an 710065, China; 210104@xsyu.edu.cn

**Keywords:** enhanced oil recovery, water plugging, high strong polymer gel, graphite powder, breakthrough pressure

## Abstract

It is difficult to plug the fracture water channeling of a fractured low-permeability reservoir during water flooding by using the conventional acrylamide polymer gel due to its weak mechanical properties. For this problem, micron graphite powder is added to enhance the comprehensive properties of the acrylamide polymer gel, which can improve the plugging effect of fracture water channeling. The chemical principle of this process is that the hydroxyl and carboxyl groups of the layered micron graphite powder can undergo physicochemical interactions with the amide groups of the polyacrylamide molecule chain. As a rigid structure, the graphite powder can support the flexible skeleton of the original polyacrylamide molecule chain. Through the synergy of the rigid and flexible structures, the viscoelasticity, thermal stability, tensile performance, and plugging ability of the new-type gel can be significantly enhanced. Compared with a single acrylamide gel, after adding 3000 mg/L of micrometer-sized graphite powder, the elastic modulus, the viscous modulus, the phase transition temperature, the breakthrough pressure gradient, the elongation at break, and the tensile stress of the acrylamide gel are all greatly improved. After adding the graphite powder to the polyacrylamide gel, the fracture water channeling can be effectively plugged. The characteristics of the networked water flow channel are obvious during the injected water break through the gel in the fracture. The breakthrough pressure of water flooding is high. The experimental results are an attempt to develop a new gel material for the water plugging of a fractured low-permeability reservoir.

## 1. Introduction

The Yanchang oilfield is a typical low-permeability oil reservoir with dense matrix pores and widespread natural micro-fractures. At the early stage of development, most oil wells undergo hydraulic fracturing to increase oil production, resulting in complex fracture networks within the reservoirs. However, as water injection operations deepen and injection-production schemes are adjusted, the injected water can flow through the fractures due to the high conductivity, leading to water breakthrough in the corresponding oil wells [1,2,3,4,5]. To address this issue, using gel to plug the fracture water channeling has been considered the most reliable measurement for the efficient development of such reservoirs [6,7,8,9,10]. Due to the strong and large-scale flow capacity of fractures, combined with the dense matrix and high injection water pressure, it requires a high strong gel with good mechanical properties to effectively plug the fracture water channeling of a fractured low-permeability reservoir [11,12].

Presently, the underground cross-linked acrylamide polymer gel system is the most widely used water plugging agent, known for its mature technology, low cost, and safety [11,13,14,15]. This system (gelant) has a low initial viscosity under surface conditions, so it has a good injection performance. After entering the reservoir, the gelant undergoes a cross-linking reaction under the reservoir conditions to form a three-dimensional network structure so that it has a plugging ability [16,17]. After that, the gelant becomes a continuous and integral gel, which plays a role of water plugging. Nevertheless, the concentration of the acrylamide polymer gel system is generally low, and the mechanical strength of the formed gel is weak. The injected water easily breaks through the gel and forms a water channeling zone again in the fracture water channeling during the subsequent water flooding process. Although continuing to increase the concentration of the acrylamide polymer gel system can improve the mechanical strength of the formed gel, it leads to a significant increase in the initial viscosity of the system and the costs, which can cause difficulties in mixing during on-site liquid preparation, an inability to complete pumping operations, or pump operation damage [18,19,20]. Therefore, increasing the mechanical strength of acrylamide-based gels is one of the key factors in improving their effectiveness in plugging fracture water channeling.

Based on the size effect and surface/interface effects of micro-materials, the overall performance of composite gels can be significantly enhanced through the physical-chemical actions occurring during the process of adding micro-materials (such as coal fly ash, nano-SiO_2_, clay mineral, etc.) to the gels [21,22,23,24]. However, there are some shortcomings for these gel systems. For example, the cost of nanomaterials is high. The performance of clay mineralized composite gel is not significantly improved. Therefore, it is necessary to develop new additives to improve the performance of polymer gel. In recent years, graphite materials have attracted much attention as a hot topic in the field of micro-materials [25,26,27,28,29]. The modified hydrophilic micron graphite powder has a layered structure with abundant hydroxyl and carboxyl functional groups on its surface, so it can be uniformly dispersed in water. An excellent material can be formed after the micron graphite powder is compounded with water-soluble polymers, and it is commercially available at a low price [30,31,32,33,34,35]. Therefore, it is technically and economically feasible to enhance the performance of polymer gels by adding micron graphite powder. In this study, by evaluating the influence of micron graphite powder on gel performance and characterizing the features of such composite gels for plugging the fracture water channeling, an attempt is made to develop a new technical system for water shut-off operations in fractured low-permeability reservoirs.

## 2. Results and Discussion

### 2.1. Effect of Micron Graphite Powder on the Structure of Polymer Gel

**Figure 1:**
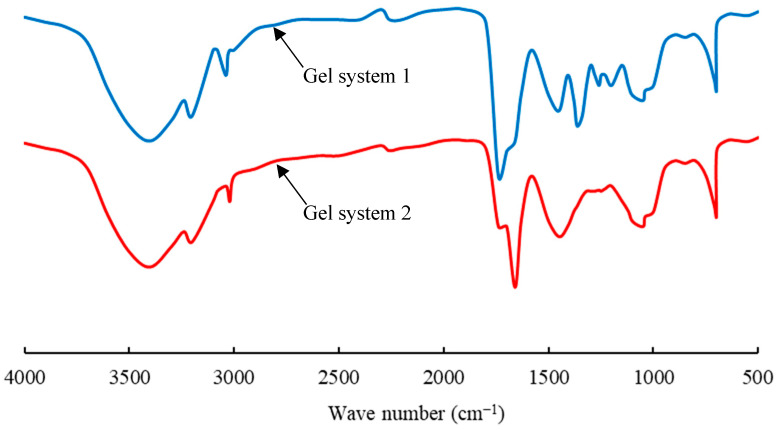
Infrared spectra of the two gels.

**Figure 2:**
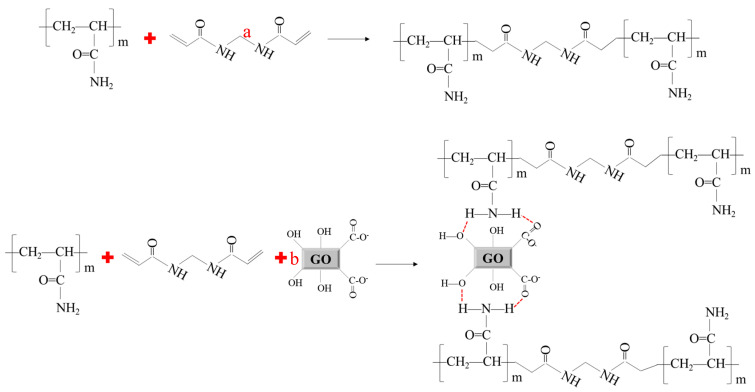
Mechanism of the cross-linking reaction of the two gels ((a): gel system 1; (b): gel system 2).

The infrared spectra of the two gel systems are shown in Figure 1. A comparison reveals that the micron graphite powder has a significant impact on the structure of acrylamide-based gels. In the spectrum of gel system 2, the characteristic absorption peaks of N–H groups are observed at 3400 cm^−1^ and 3200 cm^−1^. The stretching vibration peaks of carbonyl (C=O) groups and the bending vibration peak of N–H are observed at 1740 cm^−1^ and 1660 cm^−1^, respectively. The vibration peak at 1450 cm^−1^ corresponds to methylene (CH_2_) groups, and the stretching vibration peak of C–N is observed at 1365 cm^−1^. Comparing the spectrum of gel system 2, there are shifted peaks at 1740 cm^−1^ and 1660 cm^−1^ and different peaks at 1450 cm^−1^ and 1365 cm^−1^. This shows that there is a physical and chemical reaction between the graphite powder and the polymer [36,37,38,39]. The principle of this process can be illustrated in Figure 2. It can be observed that the interaction between the abundant hydroxyl and carboxyl groups in the layered structure of micron graphite powder and the amide groups in the polyacrylamide chain occurs, so the gel has a stronger structure.

### 2.2. Effect of Micron Graphite Powder on the Viscoelasticity of Gels

**Figure 3:**
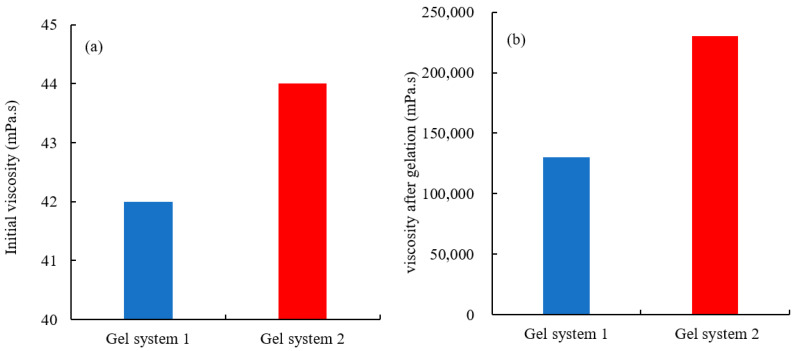
Viscosity of the two gels ((**a**): initial viscosity; (**b**): gel viscosity).

The impact of the micron graphite powder on the viscosity of the gelant before and after gelation is shown in Figure 3. After the addition of graphene oxide, the initial viscosity of the gel solution remains almost unchanged, indicating that the addition of micron-sized graphite powder has little effect on the flow properties of the gelant solution. After gelation, the viscosity of the gel is increased from 13 × 10^4^ mPa·s to 23 × 10^4^ mPa·s, indicating that graphene oxide can significantly enhance the strength of the gel.

**Figure 4:**
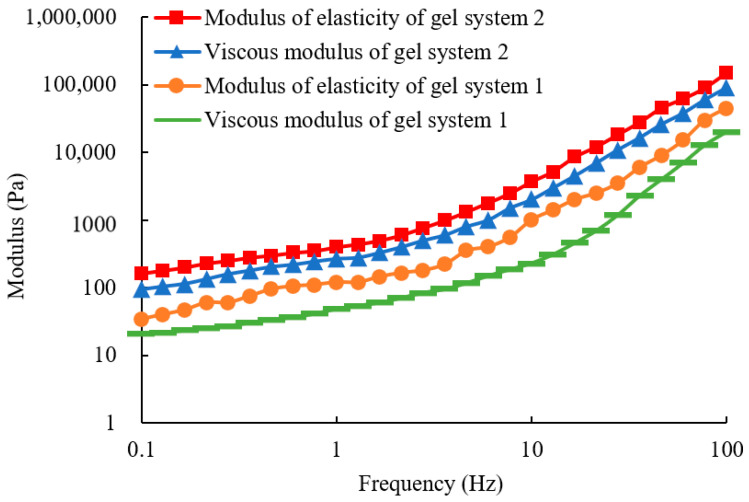
Viscoelasticity of the two types of gels.

The impact of micron graphite powder on the viscoelasticity of the gel is shown in Figure 4. After the addition of micron graphite powder, both the elastic modulus and the viscous modulus of the gel are significantly increased, indicating a substantial improvement in the overall strength of the gel. For example, at 1 Hz, the elastic modulus is increased from 120 Pa to 400 Pa, and the viscous modulus is increased from 48.6 Pa to 268 Pa. Combined with the analysis of the infrared spectra, it can be inferred that the cross-linking density of the gel is enhanced due to interactions with the amide groups on the polyacrylamide chains. During the chemical reaction, the flexible acrylamide chains can form a three-dimensional network structure through copolymerization and cross-linking reactions. The added micron graphite powder can provide a rigid structure that can support the flexible framework of the original gel acrylamide chains rather than just acting as a physical filler. The synergistic effect between the rigid and flexible structures can greatly enhance the viscoelasticity of the gel.

### 2.3. Effect of Micron Graphite Powder on the Thermal Stability of Gels

**Figure 5:**
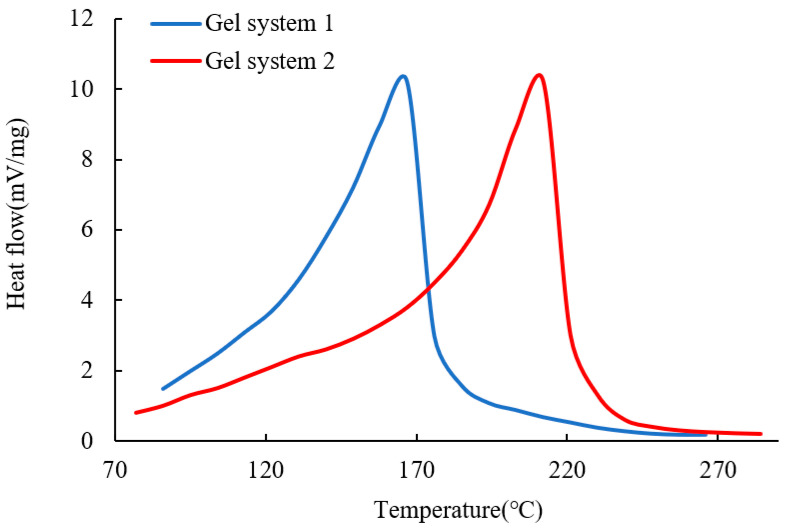
DSC curves of the two types of gels.

The heat flow curves (DSC) of the two gel systems during the heating process are shown in Figure 5. In Figure 5, it can be observed that the phase transition temperature for gel system 1 is 167 °C, while it is 212 °C for gel system 2. A comparison reveals that the addition of micron graphite powder can significantly enhance the heat resistance of the gel. When the liquid water in the gel system transitions to gaseous water, the water molecules not only need to overcome the attractive forces between other water molecules but also break free from the constraints of the gel network structure. When the network structure of the gel remains stable, it is difficult for water molecules to escape due to capillary forces. As the temperature is increased, the network structure of the gel is disrupted, and water molecules can be rapidly detached from the gel, resulting in a significant increase in the heat flow rate. Comparing the temperature points corresponding to the rapid rise in the heat flow rate, gel system 2 reaches around 170 °C, while gel system 1 reaches around 120 °C, indicating that the addition of micron graphite powder can greatly improve the heat resistance of the gel. The reason for this improvement lies in the fact that the added micron graphite powder participates in the gel formation process, leading to a denser three-dimensional network structure. Moreover, the micron graphite powder itself is an excellent heat-resistant material. Additionally, the surface effects and small size effects of the micron materials further enhance the firm binding of water molecules, thereby providing stronger heat resistance to gel system 2.

### 2.4. Effect of Micron Graphite Powder on the Tensile Properties of Gels

**Figure 6:**
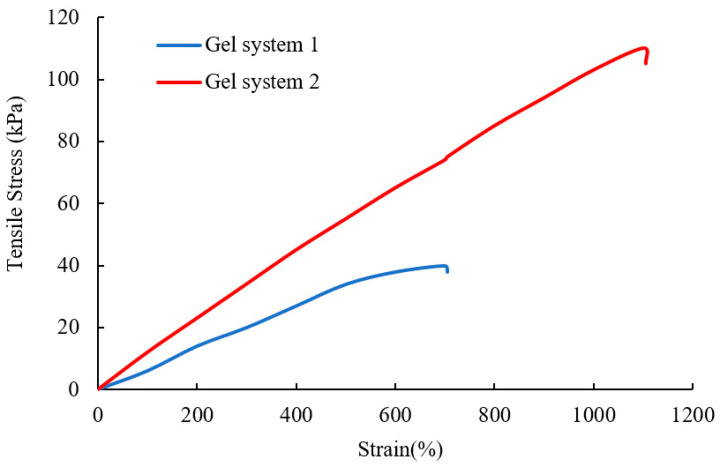
Stretching curves of the two types of gels.

The stress–strain curves of the two gels are shown in Figure 6. In Figure 6, it can be observed that gel system 1 has an elongation of 700% and a tensile stress of 38 kPa. On the other hand, gel system 2 exhibits an elongation of 1100% and a tensile stress of 110 kPa. A comparison reveals that gel system 2 has a higher tensile strength. During the chemical crosslinking process of a single acrylamide-based gel, there are many disordered crosslinking points, and the distribution of gel crosslinking points is uneven. Regions with a lower crosslinking density become weak points in the gel structure and contribute to gel rupture under tension. However, during the chemical crosslinking process of a composite gel, the micron graphite powder acts as a partial crosslinking agent, leading to an overall increase in the crosslinking density of the gel and a significant improvement in its tensile properties [40].

### 2.5. Plugging Performance of the Gel System

**Figure 7:**
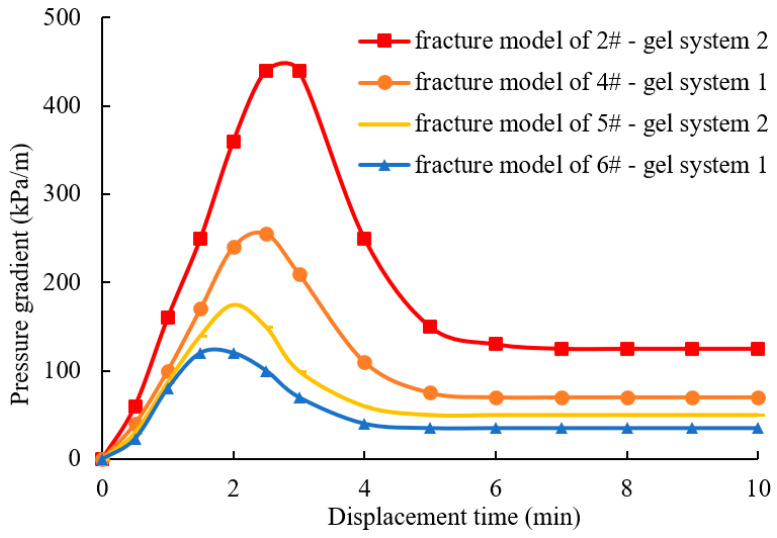
Breakthrough pressure gradient of the gel in fractures with different openings.

The breakthrough pressures of the two gels in different fractures are shown in Figure 7. After the fracture water channelings are filled with the gel, the water flow pathway is plugged, and the water injection pressure gradient is increased and then decreased until it is stabilized. The peak value of the curve represents the breakthrough pressure gradient of the gel, which indicates the plugging strength of the gel in the fracture water channeling. The stable value of the curve represents the water flow resistance after the gel is broken by the injected water, which is the residual resistance gradient of the gel in the fracture water channeling. As the aperture of the fracture is increased, both the breakthrough pressure gradient and the residual resistance gradient are both decreased. When the apertures of the fractures are 0.13 mm and 0.24 mm, the breakthrough pressure gradients of gel system 2 are 440 kPa/m and 175 kPa/m, respectively, while the breakthrough pressure gradients of gel system 1 are 255 kPa/m and 120 kPa/m, respectively. The experimental results indicate that the addition of micron graphite powder can significantly enhance the plugging strength of the gel. This is due to the fact that the micron graphite powder strengthens the gel’s viscoelasticity, tensile strength, and other properties.

**Figure 8:**
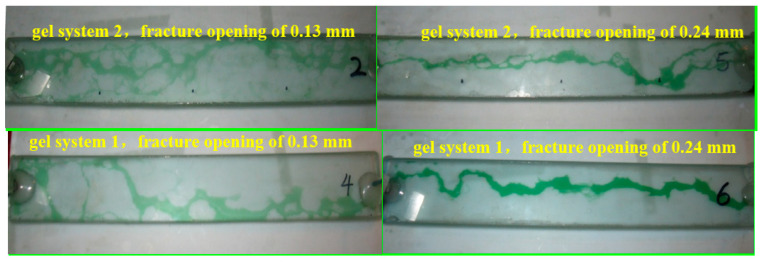
Morphology of water injection penetrating into the gel under different apertures of fracture (the green fluid is the stained water, and the transparent part is the gel).

During the subsequent water flooding process, the changes in the water injection pressure gradient are due to the breakthrough of the injected water through the gel in the fractures. The breakthrough behavior is shown in Figure 8 (the green fluid is the stained water, and the transparent part is the gel). From Figure 8, it can be observed that when the apertures of fractures are different, the penetration of injected water through the gel takes on different forms. In a smaller aperture of a fracture, such as fracture model #2, the injected water breaks through the gel and forms a mesh-like water flow channel. However, in a larger aperture of a fracture, such as fracture model #5, a strip-like water flow channel is formed. By comparing the plugging characteristics of the two in different fractures, it can be concluded that the stronger the gel, the more pronounced the mesh-like water flow channel characteristic, and the weaker the gel, the more pronounced the strip-like water flow channel characteristic. The smaller aperture of the fracture corresponds to the stronger gel, larger water injection pressure gradient, and higher energy required for water to penetrate through the gel, resulting in temporary pressure accumulation in multiple locations of the fracture, forming a mesh-like fracture. Larger fracture apertures correspond to the weaker gel and lower plugging strength, making it easier for the injected water to penetrate the weakest part of the cross-linking gel, thus forming a strip-like rupture.

**Figure 9:**
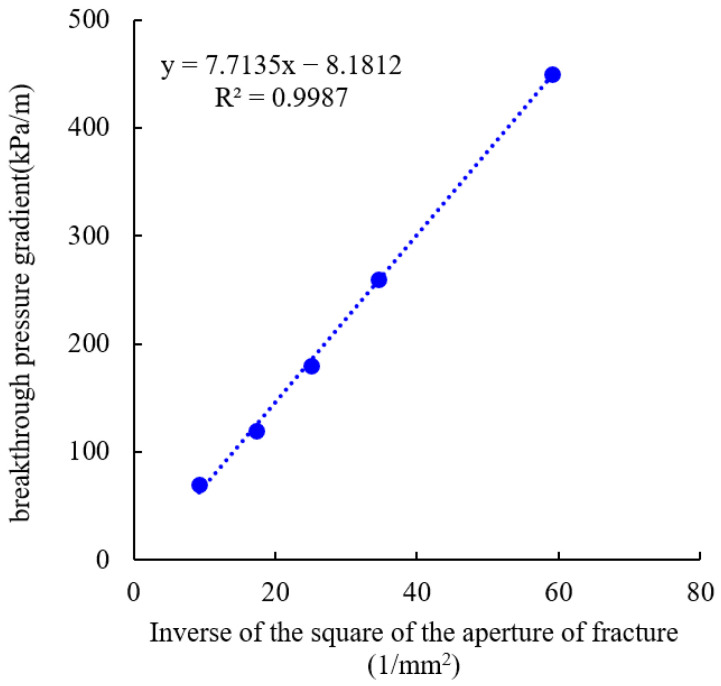
Relationship between the breakthrough pressure gradient and the inverse of the square of the aperture of the fracture.

After processing the data, it is found that the breakthrough pressure gradient is inversely proportional to the square of the aperture of the fracture, as shown in Figure 9. The aperture of the fracture has a significant impact on the plugging behavior of the gel in the fractures. Based on the results in Figure 9, the breakthrough pressure gradient of the gel can be obtained according to the size of the aperture of the fracture.

### 2.6. The Plugging Ability of the Graphene Oxide Gel System to Enhance Oil Recovery

**Figure 10:**
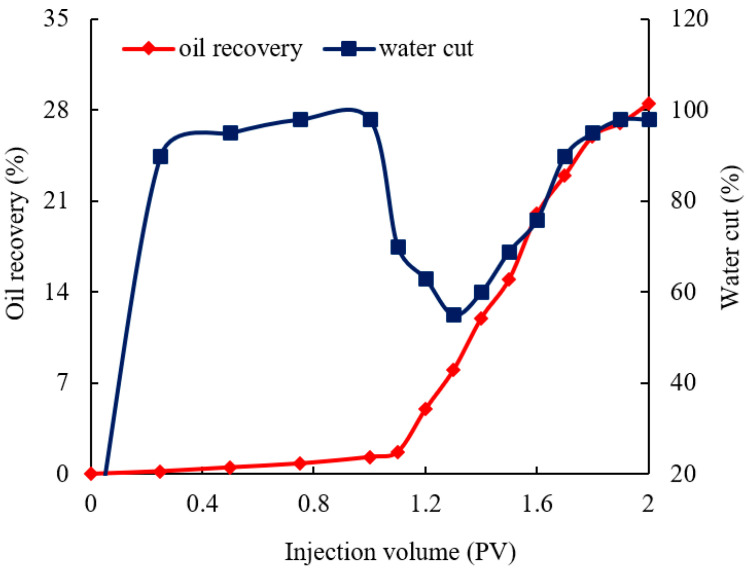
Changes in the oil recovery and water cut of the fractured core during water flooding before and after plugging.

The change in the oil recovery and water cut during plugging by using the gel in the fractured core is shown in Figure 10. Owing to the strong heterogeneity of the fracture, the water cut of the primary water flooding is almost 100%, and the oil recovery is almost minimal. After the fracture is plugged, the oil in the matrix can be displaced by the water flow. During the subsequent waterflooding, when the water cut is gradually increased to 98% again, the oil recovery is increased by 28%.

## 3. Conclusions

(1) Adding the micron graphite powder to polyacrylamide gel can significantly enhance the gel’s viscoelasticity, thermal stability, tensile strength, and plugging ability. The improvement is attributed to the interaction of the hydroxyl and carboxyl groups on the micron graphite powder layers and the amide groups on the polyacrylamide molecular chains. The micron graphite powder acts as a crosslinking agent, not only physically filling the gaps but also providing rigid support to the flexible main chains, thereby enhancing the comprehensive properties of the gel. Adding 3000 mg/L of oxidized graphene can greatly improve the comprehensive performance of the gel, increasing the elastic modulus from 120 Pa to 400 Pa and the viscous modulus from 50 Pa to 280 Pa under a frequency of 1 Hz. The thermal transition temperature can also be increased from 167 °C to 212 °C, and the tensile stress can be increased from 40 kPa to 110 kPa.

(2) During the process of plugging a fracture, when the gel is stronger, a network-like water flow channel is formed, while a strip-like water flow channel is formed when the gel is weaker. By using the micron graphite powder composite gel for plugging the fracture, the characteristics of a network-like water flow channel become evident, indicating a high plugging strength. In field construction, the stronger composite gel plugging agents can be used as segment plugs, resulting in lower implementation costs and higher economic feasibility. During the process of plugging a fracture, the breakthrough pressure gradient is decreased with the increase in the aperture of the fracture. The apertures of the fracture are 0.13 mm, 0.17 mm, 0.2 mm, 0.24 mm, and 0.33 mm, corresponding to breakthrough pressure gradients of 440 kPa/m, 250 kPa/m, 175 kPa/m, 120 kPa/m, and 70 kPa/m, respectively. The breakthrough pressure gradient is inversely proportional to the square of the aperture of the fracture. Different apertures of the fracture result in different water infiltration patterns through the gel. In small fractures, a network-like water channel is formed, while in larger fractures, a strip-like water channel is formed. The use of micron graphite powder in combination with traditional acrylamide-based gels can provide a strong material foundation for plugging the fractured low-permeability reservoir.

## 4. Materials and Methods

### 4.1. Materials

The reservoir water was simulated by preparing a solution of 10 g/L NaCl in tap water. All the solutions in the experiment were prepared by using simulated reservoir water. The micron graphite powder with a layered structure (containing a large number of hydroxyl and carboxyl groups on its surface) is a commercially available product provided by Nanjing Xianfeng Nano-Material Technology Co., Ltd, Nanjing, China. The content of oxygen-containing groups on the used graphite is 0.015 mmol/g. The particle size distribution of the graphite powder is 0.5–5 μm, and it has a low cost, making it suitable for industrial applications. Acrylamide (AM), N,N’-Methylenebisacrylamide, and potassium persulfate are all of analytical purity and were purchased from China National Pharmaceutical Group Chemical Reagent Co., Ltd, Beijing, China.

### 4.2. Instruments and Apparatus

A Brookfield viscometer, Brookfield company (Middleboro, MA, USA); RS-600HAAKE rheometer, Haake company (Karlsruhe, Germany); Nicolet 6700 Fourier Transform Infrared Spectrometer (FTIR), Thermo Fisher company (Waltham, MA, USA); DSC823 Differential Scanning Calorimeter, Mettler Toledo company (Zurich, Switzerland); and Anton-Paar Dynamic Mechanical Analyzer, Anton-Paar company (Graz, Austria), were used. A conventional physical simulation displacement experimental apparatus, Hai’an Petroleum Research Instruments Co., Ltd. (Hai’an, China), was used. For the plugging experiments, a visually observable fracture model was created to simulate reservoir fractures. This fracture model was formed by bonding two pieces of frosted glass along the sides by using epoxy resin adhesive. The aperture of the fracture was controlled by filling rubber bands with different sizes on the fracture surface. The parameters of the fractures used in the experiment are shown in Table 1. The temperature of the displacement experiment is 45 °C, and the injection rate of the fluid is 0.5 mL/min. The inlet of the fracture model is connected to a pressure sensor, and the inlet pressure is automatically recorded by the computer. The outlet of the fracture model is connected to the atmosphere. The pressure difference (ΔP) at both ends of the fracture model is numerically equal to the inlet pressure.

### 4.3. Experimental Methods

#### 4.3.1. Static Experiments

The composition of gel system 1 is 40,000 mg/L of acrylamide monomer, 2000 mg/L of N, N’-Methylenebisacrylamide (crosslinker), and 600 mg/L of potassium persulfate (initiator). The composition of gel system 2 is 3000 mg/L of micron graphite powder, 40,000 mg/L of acrylamide monomer, 2000 mg/L of N, N’-Methylenebisacrylamide (crosslinker), and 600 mg/L of potassium persulfate (initiator).

The gelation experiment is conducted at a temperature of 45 °C. The viscosity of the gel system is measured by using a viscometer at a shear rate of 7.34 s^−1^. The viscoelasticity of the gel system is measured by using a rheometer at different frequencies. The structure of the gel system is determined by using FTIR spectroscopy. The thermal stability of the gel system is determined by using a differential scanning calorimeter (DSC) at a heating rate of 3 °C/min. The temperature range for heating is from 50 to 800 °C, and nitrogen gas is used as the protective atmosphere. The mechanical properties of the gel system are determined by using a dynamic mechanical analyzer. Based on the results of the static tests of the two gel systems, the effect of micron graphite powder on the performance of acrylamide-based gels can be investigated.

#### 4.3.2. Dynamic Experiments

**Figure 11:**
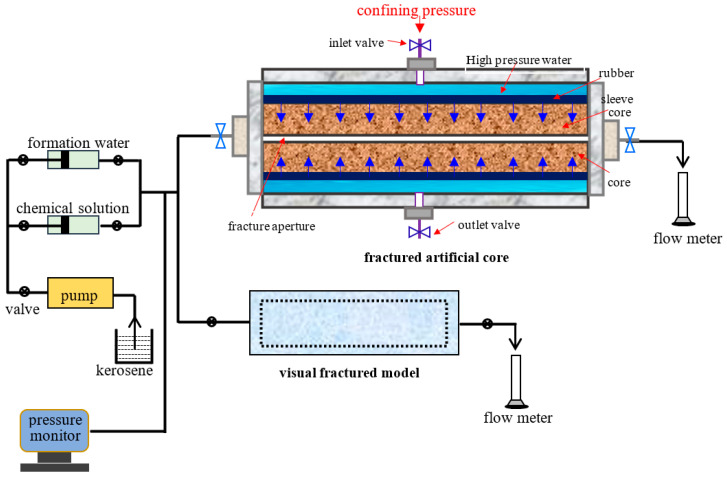
Simulation experiment device for core displacement and visual fractured model displacement [1].

The simulation experiment device for displacement is shown in Figure 11. One is the visual fracture displacement experiment. The other is the fractured core displacement experiment. During the visual fracture displacement experiment, the 1PV of gel system 1 is injected into the fracture models of #4 and #6, respectively. The 1PV of gel system 2 is injected into the fracture models of #2 and #5, respectively. Thus, after gelation, the breakthrough pressure is measured. The method of measurement is as follows. After the plugging agent in the fracture is fully gelled, the subsequent water flooding is conducted and the simulated formation water is injected into the fractures until the flow rate at the outlet is suddenly increased and the inlet pressure of the fracture is decreased. The inflection point on the injection pressure curve represents the breakthrough pressure. The experimental temperature is 45 °C and the injection rate is 0.5 mL/min. A pressure sensor is connected to the inlet of the fracture, and the inlet pressure is automatically recorded by the computer. The outlet of the fracture is exposed to atmospheric pressure, and the pressure difference ΔP between the two ends is numerically equal to the inlet pressure.

During the fractured core displacement experiment, a natural outcrop core with the size of 45 mm width × 45 mm height × 300 mm length and a water permeability of 1 × 10^−3^ μm^2^ is used to be processed into a fractured core. The aperture of the fracture is 0.24 mm. After the fractured core is saturated oil, it is displaced by the simulated water at an injection rate of 0.5 mL/min until the water cut at the outlet is up to 98%. Thus, 1 FPV of the plugging agent of the micron graphite powder–polymer composite gel is injected into the fractured core. After gelation, the subsequent water flooding is carried out again until the water cut of the effluent is 98%. Thus, the changes in the oil recovery before and after plugging can be obtained.

## Figures and Tables

**Table 1:** Parameters of fracture models.

No.	Fracture Length (cm)	Fracture Width (cm)	Diameter of Rubber Bands (Mesh Number/mm)	Fracture Aperture (mm)
#2	25	2.5	180/0.08	0.13
#4			180/0.08	0.13
#5			70/0.2	0.24
#6			70/0.2	0.24

## Data Availability

The original contributions presented in the study are included in the article, further inquiries can be directed to the corresponding authors.
